# Effects of isorhamnetin on liver injury in heat stroke-affected rats under dry-heat environments via oxidative stress and inflammatory response

**DOI:** 10.1038/s41598-024-57852-y

**Published:** 2024-03-29

**Authors:** Xinyue Yang, Hongwei Wang, Caifu Shen, Xiang Dong, Jiajia Li, Jiangwei Liu

**Affiliations:** 1 Key Laboratory of Special Environmental Medicine of Xinjiang, General Hospital of Xinjiang Military Command, Urumqi, 830000 China; 2 Shandong Provincial Third Hospital, Jinan, 25000 China; 3 Graduate School, Xinjiang Medical University, Urumqi, 830000 China

**Keywords:** Isorhamnetin, Heat shock disease, Liver injury, Oxidative stress, Inflammation, Molecular biology, Zoology, Medical research

## Abstract

Isorhamnetin is a natural flavonoid compound, rich in brass, alkaloids, and sterols with a high medicinal value. This study investigated the effects of isorhamnetin on liver injury and oxidative and inflammatory responses in heat-stroke-affected rats in a dry-heat environment. Fifty Sprague Dawley rats were randomly divided into five groups: normal temperature control (NC, saline), dry-heat control (DHC, saline), low-dose isorhamnetin-pretreated (L-AS, 25 mg/Kg), medium-dose isorhamnetin-pretreated (M-AS, 50 mg/Kg), and high-dose isorhamnetin-pretreated (H-AS, 100 mg/Kg) group. Saline was administered to the NC and DHC groups and corresponding concentrations of isorhamnetin were administered to the remaining three groups for 1 week. Blood and liver tissue were analyzed for oxidative stress and inflammation. The liver histopathological injury score, serum liver enzyme (alanine transaminase, aspartate transaminase, and lactate dehydrogenase), liver oxidative stress index (superoxide dismutase [SOD], catalase [CAT], and malondialdehyde), and inflammation index (tumor necrosis factor α [TNF-α], interleukin [IL]-1β, IL-6, and lipopolysaccharides) were significantly higher in the DHC group than in the NC group (*P* < 0.05). These index values in the L-AS, M-AS, and H-AS groups were significantly lower than those in the DHC group (*P* < 0.05). The index values decreased significantly with an increase in the concentration of isorhamnetin (*P* < 0.05), while the index values of CAT and SOD showed the opposite tendency (*P* < 0.05). The expression of liver tissue nuclear factor kappa B (NF-κB), caspase-3, and heat shock protein (HSP-70) was higher in the DHC group than in the NC group (*P* < 0.05). Comparison between the isorhamnetin and DHC groups revealed that the expression of NF-кB and caspase-3 was decreased, while that of HSP-70 continued to increase (*P* < 0.05). The difference was significant for HSP-70 among all the isorhamnetin groups (*P* < 0.05); however, the NF-кB and caspase-3 values in the L-AS and H-AS groups did not differ. In summary, isorhamnetin has protective effects against liver injury in heat-stroke-affected rats. This protective effect may be related to its activities concerning antioxidative stress, anti-inflammatory response, inhibition of NF-кB and caspase-3 expression, and enhancement of HSP-70 expression.

## Introduction

Prolonged exposure to temperatures exceeding 40 °C can disrupt the body’s natural temperature regulation, causing an imbalance that elevates the core body temperature and increases the risk of heat stroke. Heat stroke can induce a range of physiological and behavioral disorders, such as nerve damage, immune disorders, and multiple organ dysfunction^1^. The liver is involved in important functions in the body, including maintaining energy metabolism homeostasis, synthesizing bile, storing glycogen, and clearing toxins^2^. Heat stroke can cause severe liver damage. Although the precise mechanism of liver injury in heat stroke remains unclear, emerging research suggests that oxidative stress, inflammatory responses, and energy metabolism disorders are key factors^3^. Therefore, identifying substances capable of alleviating oxidative stress and inflammatory responses is important for the control and treatment of heat stroke-induced liver injury.

Bioflavonoids are the most abundant phytochemical substances in numerous fruits and vegetables^4^. These compounds exhibit a wide range of pharmacological effects, including anti-oxidative stress, anti-inflammatory response, immune regulation, anti-diabetes, anti-hypertension, and anti-cancer activities^5^. Isorhamnetin, a naturally occurring bioflavonoid, causes fewer side effects and has a higher safety profile compared with conventional drugs. Isorhamnetin can inhibit oxidative stress, ameliorate paracetamol-induced liver injury by modulating the NLRP3/NF-κB/Nrf2 pathway^6^, and mitigate oxidative stress in diabetic mice^7^. Moreover, isorhamnetin exhibits protective effects against lung injury in heat stroke-affected rats by reducing inflammatory responses and oxidative stress^8^. Additionally, isorhamnetin plays a protective role in myocardial injury induced by ischemia/reperfusion by reducing apoptosis and oxidative stress^9^. To date, the effect of isorhamnetin on liver injury in heat stroke-affected rats under dry-heat environments has not been evaluated. This study was conducted to examine the effects of isorhamnetin on oxidative stress and inflammatory responses in the liver injury of heat stroke-affected rats in a dry-heat environment and to determine the mechanism of its protective effect on the liver in a rat model with early-stage heat stroke^10^. By revealing insights into the prevention and control of environmental heat stroke, this study provides a theoretical basis for studies of ethnomedicine transformation and applications.

## Methods

### Materials

Fifty male Sprague–Dawley rats weighing 240–260 g were purchased from the Experimental Animal Center of Xinjiang Medical University (license number SCXK (Xin) 2018–0002). The rats had free access to food and water. They were housed in specific pathogen-free animal laboratories for 1 week at ambient temperature (22 ± 1 °C) and humidity (50% ± 5% relative humidity). All animal procedures were performed in strict accordance with international ethical standards and the guidelines for the care and use of experimental animals issued by the National Institutes of Health. Animal procedures were approved by the Animal Ethics Committee of the Xinjiang Military Region General Hospital (approval number: DWLL20230323) and reported in accordance with ARRIVE guidelines.

Isorhamnetin (CAT no: I811872) was provided by Shanghai Macklin Biochemical (Shanghai, China). Test kits for catalase (CAT; CAT no: A007-1-1), malondialdehyde (MDA; CAT no: A003-1), and superoxide dismutase (SOD; CAT no: A001-3) were purchased from the Nanjing Jiancheng Bioengineering Institute (Nanjing, China). Rat tumor necrosis factor (TNF)-α (CAT no: JER-06), interleukin (IL)-1β (CAT no: JER-01), and IL-6 (CAT no: JER-04) enzyme-linked immunosorbent assays (ELISA) kits were purchased from Qiaoyi Biotechnology Co., Ltd. (Anhui, China). The sodium dodecyl sulfate–polyacrylamide gel electrophoresis (SDS–PAGE) kit (CAT no: P1200) and RIPA buffer (high) (CAT no: R0010) were from Beijing Solarbio Science & Technology (Beijing, China). The BCA protein detection kit (CAT no: 23227) was purchased from Thermo Fisher Scientific, Inc. (Waltham, MA, USA). Goat anti-rabbit antibody (ab97051) was purchased from Abcam (Cambridge, UK), and antibodies against β-actin (rabbit mAb #4970), nuclear factor (NF)-κB (rabbit mAb #8242), caspase-3 (rabbit pAb #9662), and heat shock protein (HSP)-70 (rabbit pAb #4872) were purchased from Cell Signaling Technology (Danvers, MA, USA).

### Methods

After 1 week of specific pathogen-free laboratory adaptive feeding, the sample size was determined based on our previous research. Based on a study by Dong et al.^8^ of the effect of isorhamnetin on heat stroke-induced lung injury in rats, the 50 Sprague–Dawley rats were divided into the following five groups using a random number table: normal temperature control group (NC group), dry-heat control group (DHC group), low-dose isorhamnetin-pretreated group (L-AS group, 25 mg/kg), medium-dose isorhamnetin- pretreated group (M-AS group, 50 mg/kg), and high-dose isorhamnetin-pretreated group (H-AS group, 100 mg/kg). Saline was administered to the NC and DHC groups for 1 week, and the concentrations of isorhamnetin described above were administered to the other three groups. On day 8, except for rats in the NC group, all rats were transferred in The Simulated Climate Cabin for the Special Environment of Northwest Chin, deprived of food, and drinking water, and the rats were placed in the same location to ensure consistent exposure to the ambient temperature. The rectal temperature of the rats was measured 150 min after the initiation of the experiment. The rectal temperature of the DHC group reached above 40.5 °C, indicating that the dry-heat heat stroke rat model was successfully established. All rats that survived the 150-min heat exposure were included in the experiment and subjected to further analysis. After abdominal anesthesia with 3% pentobarbital, the vena cava blood was removed, the liver tissue was extracted, and blood was drawn prior to euthanasia. The serum levels of alanine aminotransferase (ALT), aspartate aminotransferase (AST), and lipopolysaccharide (LPS) were measured. Part of the liver tissue was removed for histopathological examination and electron microscopy. The remaining liver tissue was frozen in liquid nitrogen. CAT, MDA, and SOD were detected according to the kit instructions. TNF-α, IL-1β, and IL-6 levels were detected using ELISA, and HSP-70, NF-кB, and caspase-3 levels were detected using western blotting.

### Histopathology

The middle lobe of the right liver lobe was fixed in 4% paraformaldehyde for 1 week, dehydrated, paraffin embedded, sectioned 5 μm slices, and stained with hematoxylin and eosin. After staining, histopathological changes in the liver were observed under an optical microscope, and the degree of liver injury was evaluated using the liver injury scoring method^11^.

### Changes in liver tissue under electron microscopy

Kidney specimens were cut into 2 mm fragments, soaked in 2.5% glutaraldehyde overnight at 4 °C, washed three times with 0.1 M phosphate buffer, and fixed with 1% osmium tetroxide at 4 °C for 2 h. The sample was placed in increasing concentrations of ethanol for dehydration. Using epoxy resin for tissue penetration and embedding and heated at 70 °C for 9 h. Trimming and slicing the sample. The sections were stained with uranyl acetate and lead citrate at 25 °C for 15 min and observed under a transmission electron microscope (JEM-1230; JEOL, Tokyo, Japan).

### Determination of serum liver zymogram

Venous blood was centrifuged at 1500×*g* for 10 min, and the supernatant was collected. The plasma ALT, AST, and LDH levels were measured using a fully automated biochemical analyzer (XA-46000996, Shenzhen Mindary Biomedical Electronics, Shenzhen, China).

### Preparation of liver tissue homogenates

Approximately 0.1 g of liver tissue was placed in a centrifuge tube and cut into small pieces with sharp scissors. After adding 900 µL of Phosphate Buffer Solution (PBS) to each centrifuge tube, an electric homogenizer was used to grind the liver tissue and obtain 10% homogenization. The homogenate was left to stand for 2 h and centrifuged at 12,000×*g* for 10 min in a low-temperature centrifuge (Heraeus Fresco 21, Thermo Fisher Scientific); the supernatant was collected to detect inflammatory factors and oxidative stress indicators. All procedures were performed on ice.

### Determination of inflammatory factors and oxidative stress indicators

The TNF-α, IL-1β, IL-6, LPS, CAT, MDA, and SOD levels in liver tissue homogenates were determined using ELISA kits. Optical densities were read with a microplate reader (550, Bio-Rad Laboratories, Hercules, CA, USA). The data were recorded, a standard curve was plotted, and protein concentrations were calculated.

### Western blotting

The liver tissue (0.1 g) was cut with scissors, ground in RIPA buffer (0.9 mL) with an electric homogenizer, and placed on ice for 2 h. After centrifuging the homogenate at 12,000×*g* and 4 °C for 15 min, the protein concentration was measured using the BCA method. RIPA buffer was then added to balance the protein concentration in the liver tissue homogenate. All samples were mixed with 2 × loading buffer, boiled for 10 min, and separated using SDS–PAGE at 50 V for 30 min, followed by 100 V for 90 min using an electrophoresis instrument (PowerPac HC, Bio-Rad Laboratories). Proteins in the gel were transferred the protein to a polyvinylidene fluoride (PVDF) membrane for 20–40 min. The PVDF membrane was blocked with 5% skimmed milk at 25 °C ± 1 °C for 2 h; antibodies against NF-κB (1:1000), HSP70 (1:1000), caspase-3 (1:1000), and β-actin (1:1000) were added and incubated overnight at 4 °C. The membranes were washed five times with TBST (5 min each) and incubated with the corresponding second antibody (1:20,000) to 25 °C ± 1 °C. After washing the PVDF membrane three times with TBST solution, bands were visualized via chemiluminescence (ChemDoc-IT^®^510 Imager; Ultra-Violet Products Ltd., Cambridge, UK). The membranes were scanned, and band intensities were analyzed using Visionworks LS (version 8.1.2; Ultra-Violet Products Ltd.).

### Statistical analysis

Data were analyzed using SPSS 23.0 statistical software (SPSS, Inc., Chicago, IL, USA). The data were expressed as the x ± s, and one-way analysis of variance and the least significant difference test were used; a difference of *P* < 0.05 was considered to indicate a statistically significant difference.

## Results

### Rat liver structural changes and lesion histopathology score

**Figure 1:**
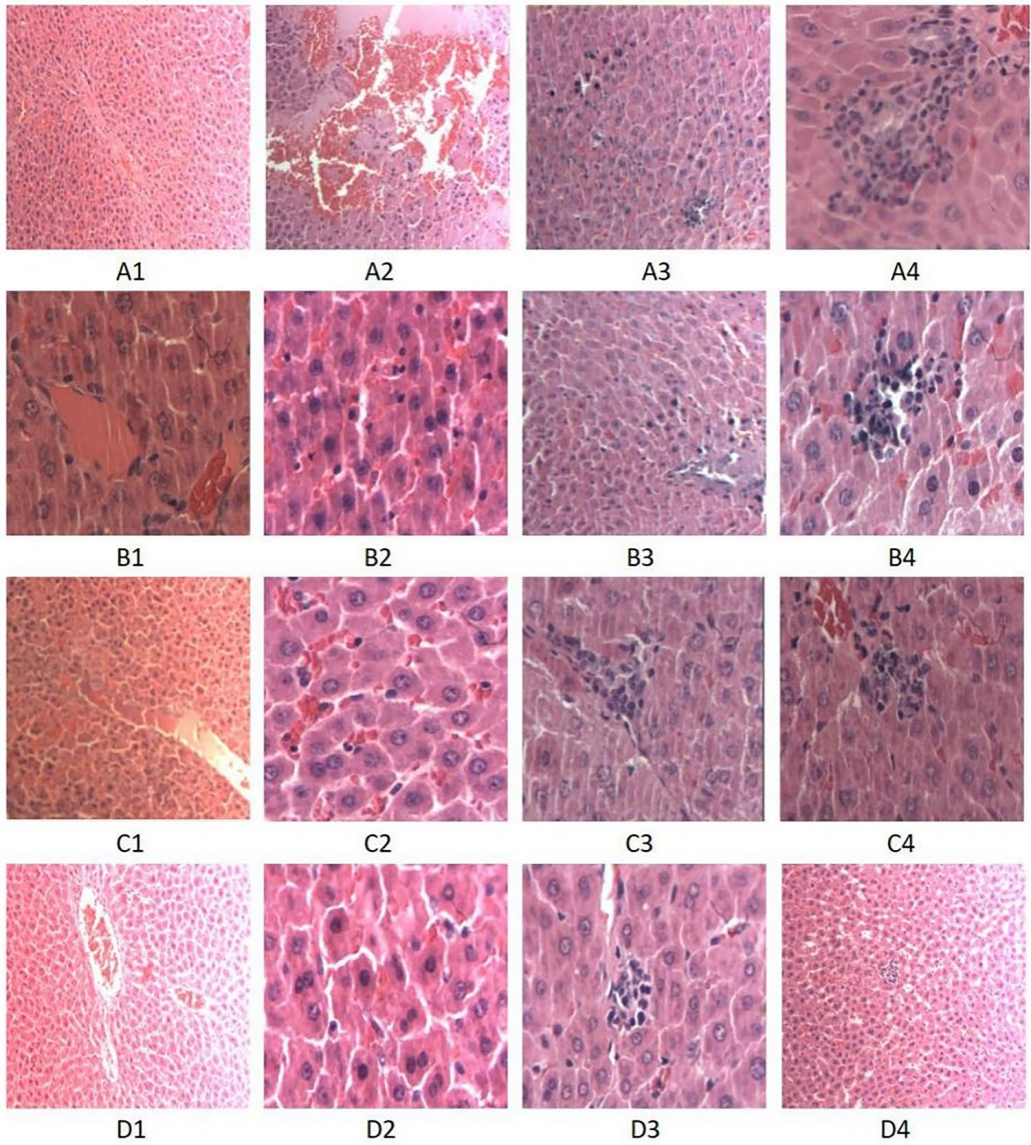
Liver structure changes in each group (HE (hematoxylin and eosin) × 100). (**A**) DHC group; (**B**) L-AS group; (**C**) M-AS group; (**D**) H-AS group.

**Figure 2:**
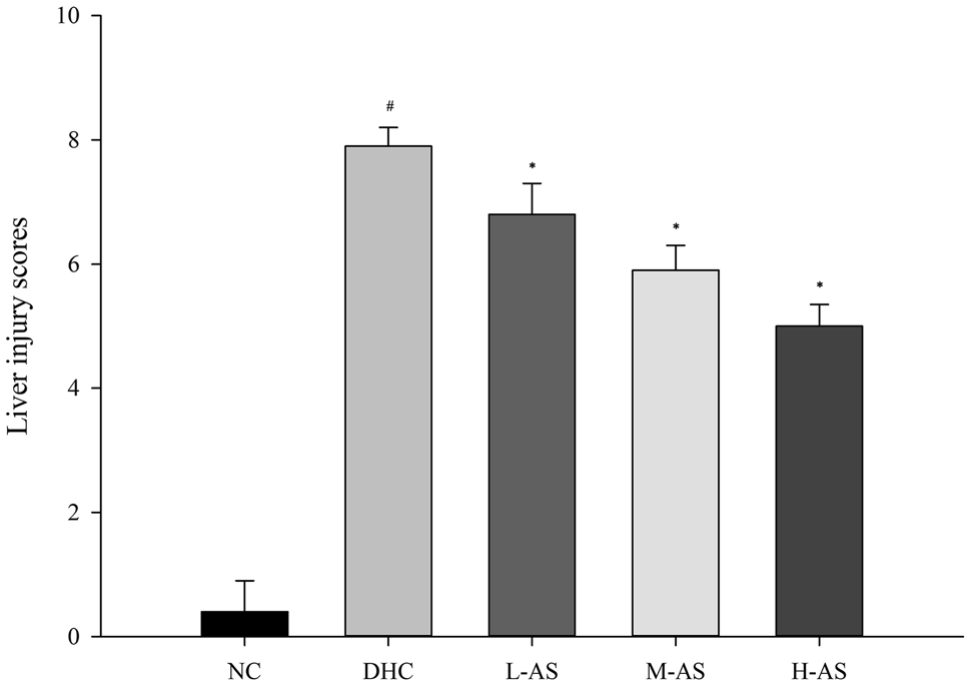
Histopathological scores of liver injury in each group. ^#^*P* < 0.05, compared with the NC group; *P < 0.05, compared with the DHC group and the pairwise comparison.

Light microscopy revealed that the hepatocyte structure of the NC group was intact. Liver cells in the DHC group showed edema, eosinophilic changes, cell death, liver cell cytoplasmic red staining, unclear cell boundary, central venous thrombosis, and other histopathological changes. The degree of histopathological hepatocyte damage across all isorhamnetin-pretreated groups was lower than that in the DHC group (Fig. 1). The histopathological injury scores of the NC, DHC, and three isorhamnetin-pretreated (L-AS, M-AS, and H-AS) groups were 0.3 ± 0.48, 7.90 ± 0.32, 6.70 ± 0.48, 5.80 ± 0.42, and 4.90 ± 0.32, respectively (Fig. 2).

### Ultrastructural changes in hepatocytes in each group

**Figure 3:**
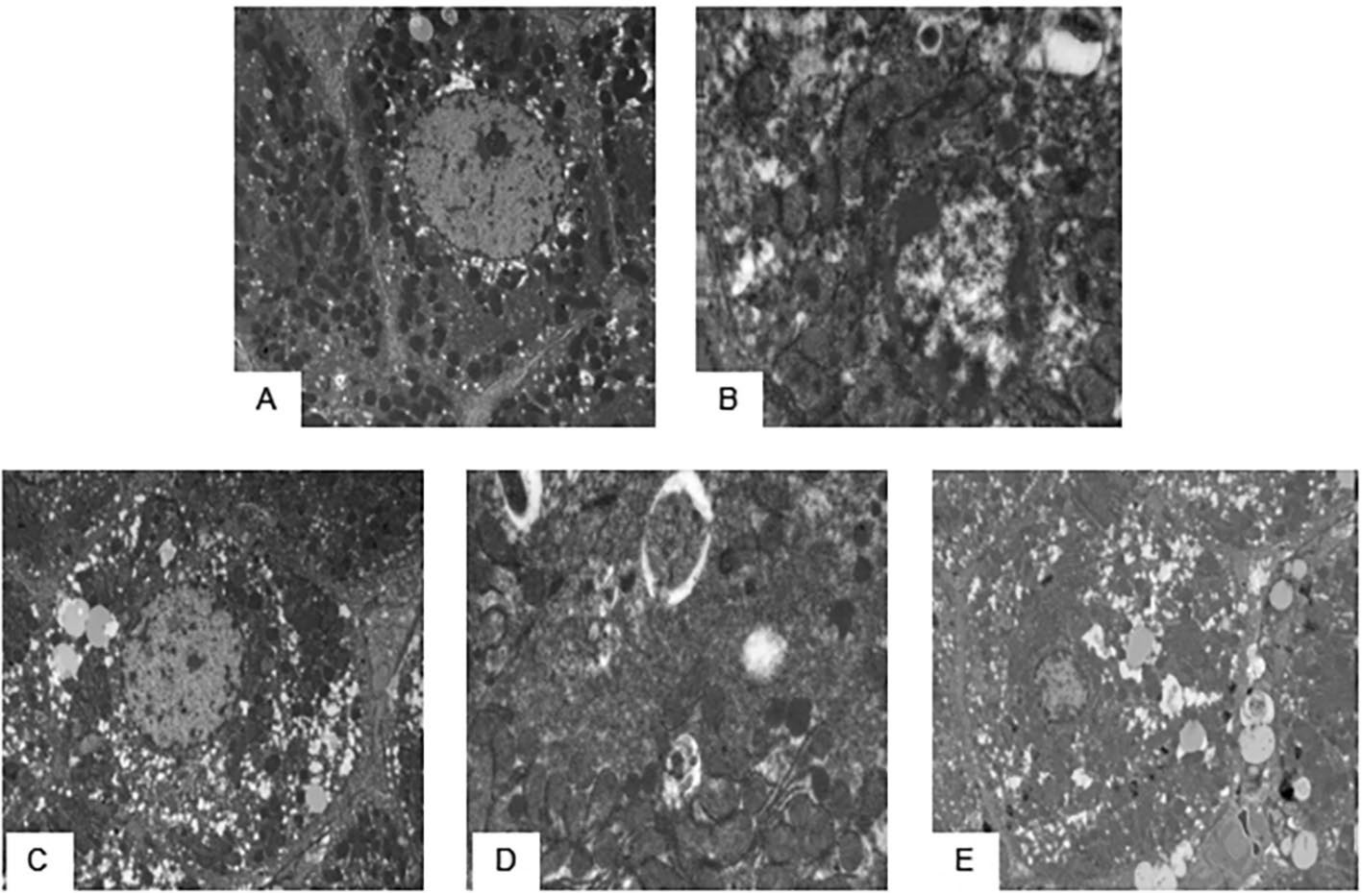
Ultrastructural changes in rat hepatocytes in each group under electron microscopy. (**A**) NC group. (**B**) DHC group. (**C**) L-AS group. (**D**) M-AS group. (**E**) H-AS group.

Electron microscopy was used to examine the liver tissue of the NC group and revealed that the cell membrane structure of the hepatocytes, the nuclear membrane, and the mitochondrial structure were intact (A). In the DHC group, the euchromatin ultrastructure of the nucleus gradually disappeared and more heterochromatin-like structures appeared; the nuclear membrane pores were blurred. The nuclear membrane was incomplete. The mitochondrial structure was disordered and disappeared. The cell membrane structure was destroyed and the cells underwent lysis or necrosis (B). In the L-AS group, there was less chromosomal heterochromatin in the nucleus, no obvious damage to the mitochondrial structure, and occasional destruction of cell membrane structure (C). In the M-AS group, there was intact nuclear structure and no obvious chromatin heterochromatin. The mitochondrial structure was clear and the cell membrane was intact (D). In the H-AS group, the hepatocyte structure was intact, the nuclear structure was intact, and mitochondria were increased (E) (Fig. 3).

### ALT and AST levels as well as MDA, SOD, and CAT contents in each group

Compared with those in the NC group, the levels of ALT, AST, and LPS in the DHC group were significantly higher (*P* < 0.05). The levels of ALT, AST, and LPS in the blood of the isorhamnetin-pretreated groups were lower than those in the DHC group (*P* < 0.05). These differences were significant across the three isorhamnetin-pretreated groups (*P* < 0.05) (Table 1).

**Table 1:** Isorhamnetin effects on liver function and oxidative stress in heat-stroke-affected rats under a dry-heat environment (x ± s).

Group	N	ALT (U/L)	AST (U/L)	MDA (nmol/mg)	SOD (U/mg)	CAT (U/mg)
NC	10	48.20 ± 5.45	175.70 ± 11.44	9.17 ± 0.59	92.04 ± 4.83	60.30 ± 2.31
DHC	10	105.10 ± 6.49^a^	345.60 ± 19.45^a^	20.98 ± 1.27^a^	35.90 ± 2.31^a^	24.27 ± 2.92^a^
L-AS	10	94.80 ± 7.58^bc^	309.30 ± 15.49^bc^	18.45 ± 0.79^bc^	39.40 ± 2.25^bc^	30.13 ± 2.06^bc^
M-AS	10	84.10 ± 4.84^bc^	277.10 ± 11.05^bc^	17.56 ± 0.69^bc^	43.90 ± 3.11^bc^	34.33 ± 1.80^bc^
H-AS	10	71.40 ± 6.23^bc^	240.90 ± 12.85^bc^	15.63 ± 0.97^bc^	49.40 ± 2.26^bc^	39.97 ± 1.68^bc^

^a^*p* < .05, compared with NC group; ^b^*p* < .05, compared with DHC group; ^c^*p* < .05, pairwise comparison of the isorhamnetin-pretreated groups.

### LPS, TNF-α; IL-1β, and IL-6 levels in each group

The levels of TNF-α, IL-1β, and IL-6 in the livers of the DHC group were higher than those in the NC group (*P* < 0.05). Compared with those in the DHC group, the levels of TNF-α, IL-1β, and IL-6 in the isorhamnetin-pretreated groups were lower (*P* < 0.05). The difference was significant across all isorhamnetin-pretreated groups (*P* < 0.05) (Table 2). In the ELISA test, all indicator tests were conducted on all samples three times independently. The inter-assay coefficient variances of the ELISA test are shown in Table 3.

**Table 2:** Effects of isorhamnetin on lipopolysaccharide (LPS), TNF-α, IL-1β, and IL-6 levels in heat-stroke-affected rats under a dry-heat environment (x ± s).

Group	N	LPS (EU/ml)	TNF-α (pg/ml)	IL-1β (pg/ml)	IL-6 (pg/ml)
NC	10	1.54 ± 0.30	4.3 ± 1.16	4.31 ± 1.25	10.1 ± 1.52
DHC	10	18.10 ± 1.44^a^	14.1 ± 1.17^a^	27.0 ± 1.66^a^	48.8 ± 2.30^a^
L-AS	10	14.20 ± 1.85^bc^	12.60 ± 1.26^bc^	23.8 ± 2.30^bc^	43.6 ± 1.70^bc^
M-AS	10	12.60 ± 1.90^bc^	9.60 ± 1.17^bc^	19.7 ± 2.00^bc^	38.82 ± 2.58^bc^
H-AS	10	7.39 ± 1.28^bc^	7.00 ± 1.49^bc^	14.8 ± 2.09^bc^	34.9 ± 2.02^bc^

^a^*p* < .05, compared with the NC group; ^b^*p* < .05, compared with DHC group; ^c^*p* < .05, pairwise comparison of the isorhamnetin-pretreated groups.

**Table 3:** The inter coefficient variances of the ELISA test.

Index (%)	MDA (nmol/mg)	SOD (U/mg)	CAT (U/mg)	LPS (EU/ml)	TNF-α (pg/ml)	IL-1β (pg/ml)	IL-6 (pg/ml)
Inter coefficient variances	8.82	6.87	8.81	9.03	8.93	12.41	8.93

### Western blotting to detect HSP-70; NF-кB, and caspase-3 levels in the liver tissue

**Figure 4:**
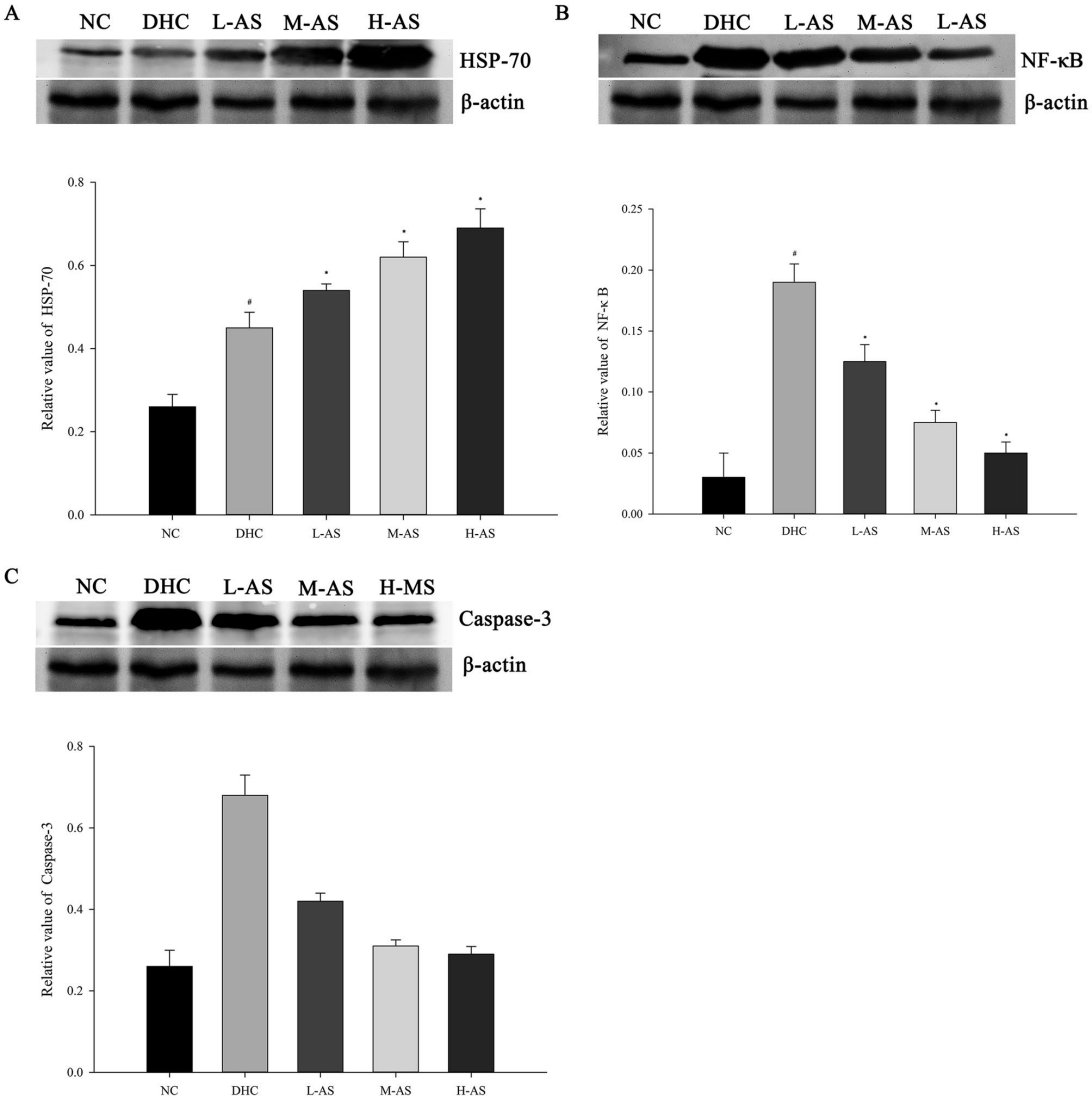
Western-blotting to detect the levels of HSP-70, NF-кB, and caspase-3 in the liver tissue. (**A**) ^#^*p* < .05, compared with NC group; *p < .05, compared with DHC group and the pairwise comparison. (**B**, **C**): ^#^*p* < .05 compared with NC group; *p < .05, compared with DHC group and L-AS vs H-AS.

The expression of HSP-70, NF-ĸB, and caspase-3 was significantly higher in the DHC group than in the NC group (*P* < 0.05). Compared with that in the DHC group, the expression levels of NF-ĸB and caspase-3 were significantly lower after treatment with isorhamnetin, and the expression of HSP-70 was significantly lower in the H-AS group than that in the L-AS group (*P* < 0.05). The protein expression levels of HSP-70 were significantly higher after treatment with isorhamnetin (*P* < 0.05), with significant differences across all isorhamnetin-pretreated groups (*P* < 0.05) (Fig. 4).

## Discussion

Heat stroke refers to a disruption in the regulation of body temperatures induced by high temperature and dry environments. In this condition, heat accumulation surpasses the body’s ability to dissipate it, resulting in varying degrees of impairment to cellular and multiple organ function, which can be life-threatening^12^. The pathogenesis of heat stroke disease remains poorly understood. Available evidence suggests that heat stroke is often caused by heat stress resulting from exposure to high temperatures or strenuous exercise. The key mediators of heat stress activation are the pro-inflammatory cytokine cascade and oxidative stress^13^. Animal experiments showed that direct heat damage, the inflammatory response, and oxidative stress can cause liver damage and, in severe cases, liver cell necrosis^11,14,15^. Isorhamnetin are natural flavonoids widely found in plants and exert antioxidant, anti-inflammatory, anti-mutagenic, and anti-cancer abilities, which regulate key cellular enzyme functions^5^. Our study confirms the above research results. We analyzed the characteristics of liver injury caused by heat stroke and found that isorhamnetin protects against liver injury in heat stroke-affected rats. This protective effect may be related to the anti-oxidative stress and anti-inflammatory effects of isorhamnetin and involved inhibition of NF-кB and caspase-3 expression and enhancement of HSP-70 expression.

We observed that heat radiation not only liver caused tissue damage but also led to alterations in the ultrastructure of the liver. In the isorhamnetin-pretreated groups of rats, liver tissue damage decreased with increasing doses of isorhamnetin, suggesting that isorhamnetin protects against liver damage. Various transaminases are present at different levels in different tissue types. Hepatocytes contain large amounts of ALT and AST. When liver cells are stimulated and damaged, ALT and AST are released into the bloodstream^16^. Hence, monitoring of blood ALT and AST levels serves as an important indicator for evaluating liver function. Heat stroke often triggers fluctuations in ALT and AST levels^17^. In the DHC group, the levels of ALT and AST significantly increased, indicating liver damage in these rats. Although the isorhamnetin-pretreated groups also exhibited elevated ALT and AST levels, the increases were not significant compared with those in the DHC group. Thus, isorhamnetin can reduce liver damage, thereby reducing ALT and AST levels in the bloodstream.

During heat stroke, the body generates large quantities of highly reactive molecules, such as reactive oxygen species (ROS) and reactive nitrogen species, disturbing the balance of thermal oxidative stress. This disruption leads to direct and indirect damage to liver cells. ROS can damage macromolecules such as proteins, lipids, and DNA by altering their structures and functions, thus impacting overall cellular function^18^. Additionally, extracellularly produced ROS can induce protease damage. Anti-proteases are proteins that function to control and regulate proteolytic enzymes. Damage to the protease barrier not only disrupts the protease-antiprotease balance but also promotes tissue damage through uncontrolled proteolysis at the injury site. Furthermore, most of the body’s redox reactions occur in the mitochondria. Excessive ROS production can damage the mitochondrial membrane, impairing cellular energy supply and resulting in liver cell damage.

In addition, the accompanying increase in activation of the mitochondrial apoptosis pathway leads to apoptosis^19^. MDA, commonly used as an indicator of lipid peroxidation, reflects ROS levels^20^. Antioxidant enzymes such as SOD and GSH scavenge ROS^21^, but elevated ROS levels cause peroxidation of lipids and other molecules. SOD and CAT protect cells from oxidative stress by detoxifying carcinogens or reducing stress; overproduction of ROS alters the oxidant‐antioxidant balance. Excessive ROS disrupts the membrane lipid composition through lipid peroxidation, consequently increasing the levels of MDA, a final metabolite product of lipid peroxidation^22^. The peroxidation reaction increases free radical production, exacerbating cell damage. The main function of this reaction is to catalyze the decomposition of H_2_O_2_ into H_2_O and O_2_, preventing H_2_O_2_ from reacting with O_2_ under the action of iron chelate to form harmful –OH^23^.

We observed significant decreases in CAT and SOD levels of the DHC group and a significant increase in the MDA content in the liver tissues compared to those in the NC group, indicating increased oxide and antioxidant levels in response to heat radiation. This increase in oxidative stress, reflected by increased MDA levels, indirectly reflects elevated ROS levels in the liver. In contrast, CAT and SOD levels increased, whereas the MDA content decreased, in the liver tissues compared with those in the DHC group. Moreover, with increasing doses of isorhamnetin, CAT and SOD levels increased and the MDA content decreased in the liver tissues of the isorhamnetin-pretreated group; the difference between groups was significant. These results indicate that isorhamnetin attenuated the oxidative stress response of the liver, thereby reducing liver damage.

HSP-70 is a molecular chaperone that is constitutively expressed under normal conditions to maintain protein homeostasis and is induced during environmental stress^24,25^. HSP-70 interacts with unfolded proteins to prevent irreversible polymerization and catalyze refolding of its substrates in an ATP- and co-chaperone-dependent manner^26^. The HSP-70 family, which acts on various substrates, including newly synthesized and denatured proteins, combines with other chaperones to stabilize existing proteins against aggregation and facilitate the folding of newly translated polypeptides in the cytosol and organelles^27^. These chaperones recognize non-native conformations of proteins, contributing to these processes. Beyond its chaperone activity, HSP-70 is important for the maturation and inactivation of nuclear hormones and other signal transduction molecules. Under high-temperature and other stimulating conditions, HSP-70 expression in the body significantly increases, enhancing the body’s ability to withstand heat and stress^28^, which aligns with our findings.

In the DHC group, HSP-70 expression in the liver tissue was increased, likely induced by self-protection mechanisms in response to stress. We also observed significantly increased HSP-70 expression in the isorhamnetin-pretreated groups, suggesting that isorhamnetin exerts a protective effect on the rat liver by increasing HSP-70 expression in the liver. With increasing isorhamnetin doses, HSP-70 expression increased. However, once HSP-70 expression reached a specific threshold, increasing the isorhamnetin dosage did not significantly increase HSP-70 expression.

Following exposure to heat radiation, the damage caused by inflammatory reactions on tissues to organisms becomes notable. TNF-α, produced by numerous immune cells including T cells, B cells, natural killer cells, and macrophages^29^, mediates cellular responses via interactions with TNF-R1 and TNF-R2 receptors, activating apoptotic pathways depending on the cell type and physiological background^30,31^. TNF-α plays a key regulatory role in inflammation and host defense against bacterial infections^32^. Furthermore, TNF-α damages endothelial cells in blood vessels, leading to vascular dysfunction, thrombosis, and local blood flow blockage in tissues, thereby causing hemorrhage and hypoxic necrosis^33^. TNF-α also serves as an endogenous pyrogen, stimulating acute phase protein synthesis in hepatocytes under heat stress and inducing IL-6 production in other cells^33^. Our results confirm those of previous studies; the levels of TNF-α, IL-1β, and IL-6 in the liver tissue of rats in the DHC group were significantly increased. In contrast, the levels of TNF-α, IL-1β, and IL-6 in the isorhamnetin-pretreated group were lower than those in the DHC group, indicating that isorhamnetin can reduce the expression of these inflammatory factors, subsequently inhibiting some cascades reducing liver tissue damage.

In addition, TNF-α activates the expression of upstream NF-кB^34^, a multi-effect transcription factor present in almost all cell types and involved in various biological processes such as inflammation, immunity, differentiation, cell growth, tumorigenesis, and apoptosis. NF-кB plays a complex and important role in the regulation of the immune response and inflammation^35^. NF-κB can function as an inflammatory factor, endotoxin, and oxidative stress factor and is an important mediator of oxidative stress^36^. Our research findings also indicate significantly increased NF-κB expression in the liver of the DHC group compared with that in the NC group, indicating that heat stroke caused inflammatory responses in the body in a process involving TNF-α and IL-6. These elevated levels confirmed that inflammatory factors and upstream factors participate in the early and intermediate stages of heat stroke-induced inflammatory responses, triggering cascades that aggravated tissue damage. Additionally, NF-κB expression was decreased in the isorhamnetin-pretreated groups compared with that in the DHC group, suggesting isorhamnetin’s capacity to inhibit NF-κB expression and the ability of NF-κB to regulate inflammatory factor expression, thus suppressing the inflammatory response.

Whether through thermal direct damage, inflammatory reactions induced by internal environment changes, or oxidative stress responses, the result is liver cell damage. To maintain internal environment stability and reduce damage, the body actively undergoes apoptosis, a strictly regulated mode of cell suicide characterized by nuclear pyknosis, cell shrinkage, cell membrane blebbing, and DNA fragmentation^37^. Caspases form a family of cysteine proteases that are central regulators of apoptosis. Inflammatory factors and pro-apoptotic stimuli activate the promoter (caspase-11), which in turn activates caspase-1, which functions directly with caspase-3 to promote cellular inflammatory responses and apoptosis. Caspase-3 is a reliable indicator of apoptosis^38–40^ and an important peripheral and intrinsic apoptotic pathway for the activation of apoptotic proteases^41^. In agreement with these findings, we observed increased caspase-3 expression in the rat liver tissue in the DHC group, indicating liver tissue apoptosis due to heat radiation. In contrast, caspase-3 expression in the isorhamnetin-pretreated groups was lower than that in the DHC group at high doses of isorhamnetin. This may be because isorhamnetin inhibits the expression of caspase-3 or inhibits a factor upstream of the caspase family, or because isorhamnetin reduces inflammatory responses and caspase family activation by inflammatory factors. These factors induce the expression of caspase-3, which plays a role in liver protection.

The liver exhibits a close relationship with the intestines, and the role of “intestinal-hepatic axis” should be considered. Previous studies have demonstrated that heat stroke changes intestinal mucosa permeability, causing mucosal barrier damage^42–44^. Subsequently, intestinal bacteria entering the bloodstream through the portal vein induce flora shifts, leading to bacteremia, sepsis, and systemic inflammatory response syndrome, ultimately resulting in multiple organ dysfunction syndrome that directly threatens life^43,45^. Various inflammatory factors and endotoxins can also activate NF-κB, triggering amplification of cascade reactions^46^; combined with our results, isorhamnetin may protect intestinal mucosa permeability.

## Conclusion

This study provides a theoretical basis for effective prevention and control of heat and disease in desert dry-heat environments and provides a foundation for the application of ethnic medicine to new fields of research. However, further research is needed to elucidate the potential mechanisms and practical significance of using isorhamnetin to treat heat stroke and related diseases.

### Supplementary Information

Supplementary Figure 1.

Supplementary Figure 2.

Supplementary Figure 3.

Supplementary Figure 4.

## Data Availability

The datasets used and/or analyzed during the current study are available from the corresponding author on reasonable request.
