# Supplementary figures and images for: Effects of isorhamnetin on liver injury in heat stroke-affected rats under dry-heat environments via oxidative stress and inflammatory response
Source: Sci Rep. 2024 Mar 29;14:7476. doi: 10.1038/s41598-024-57852-y (PMC10980765; doi:10.1038/s41598-024-57852-y)

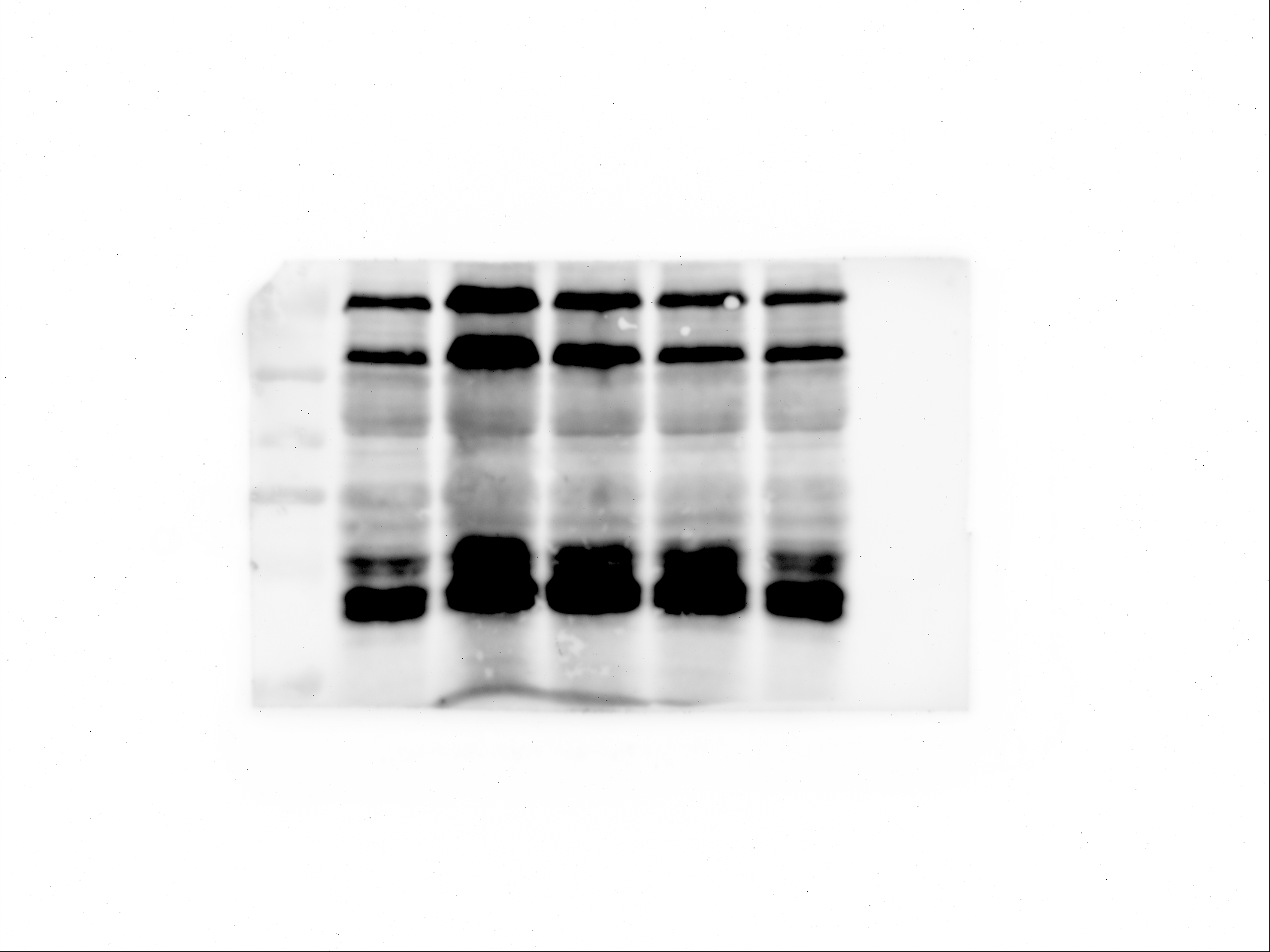

Supplement: Supplementary file 1 — Supplementary Figure 1. [file 41598_2024_57852_MOESM1_ESM.tif]

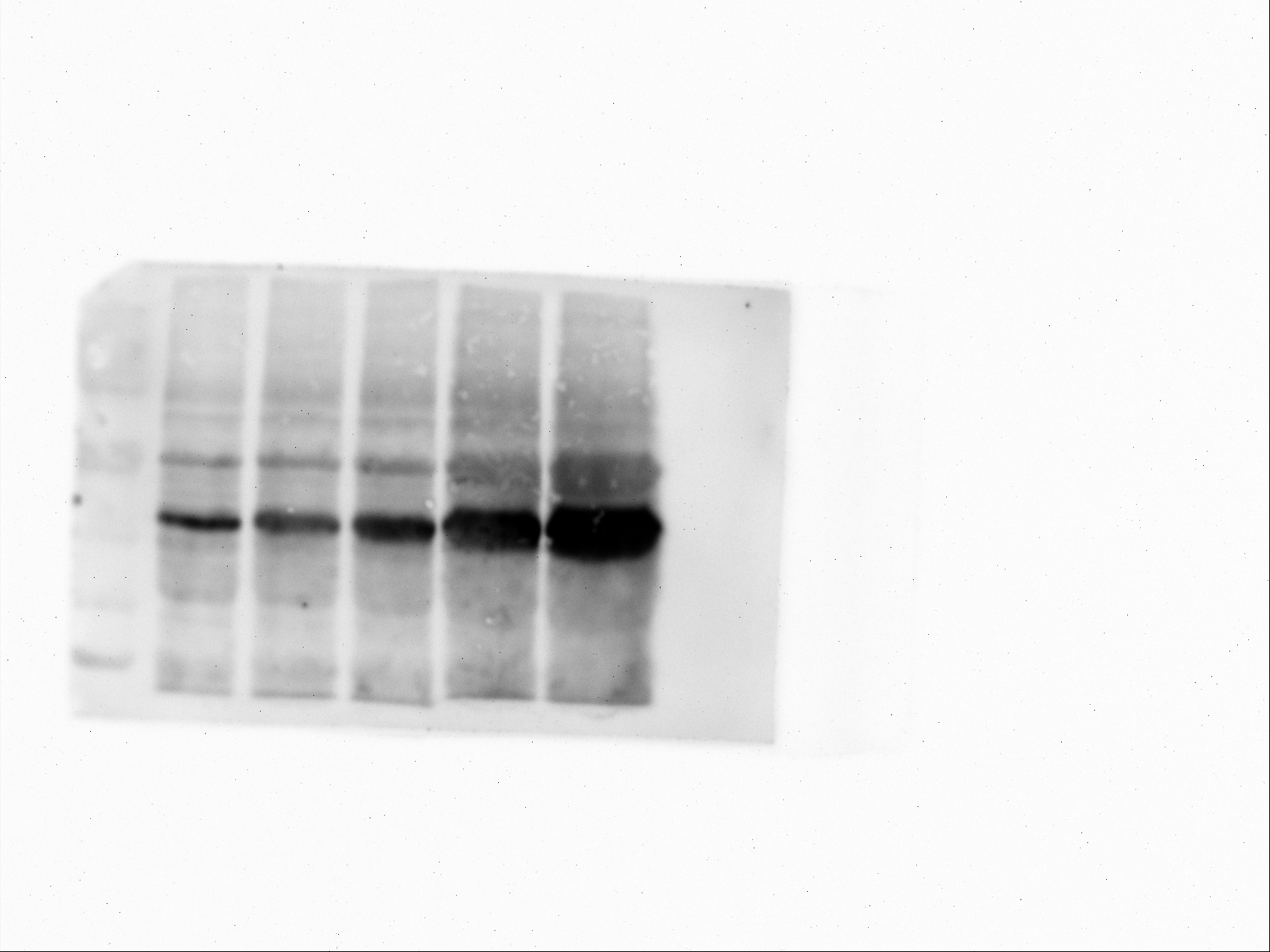

Supplement: Supplementary file 2 — Supplementary Figure 2. [file 41598_2024_57852_MOESM2_ESM.tif]

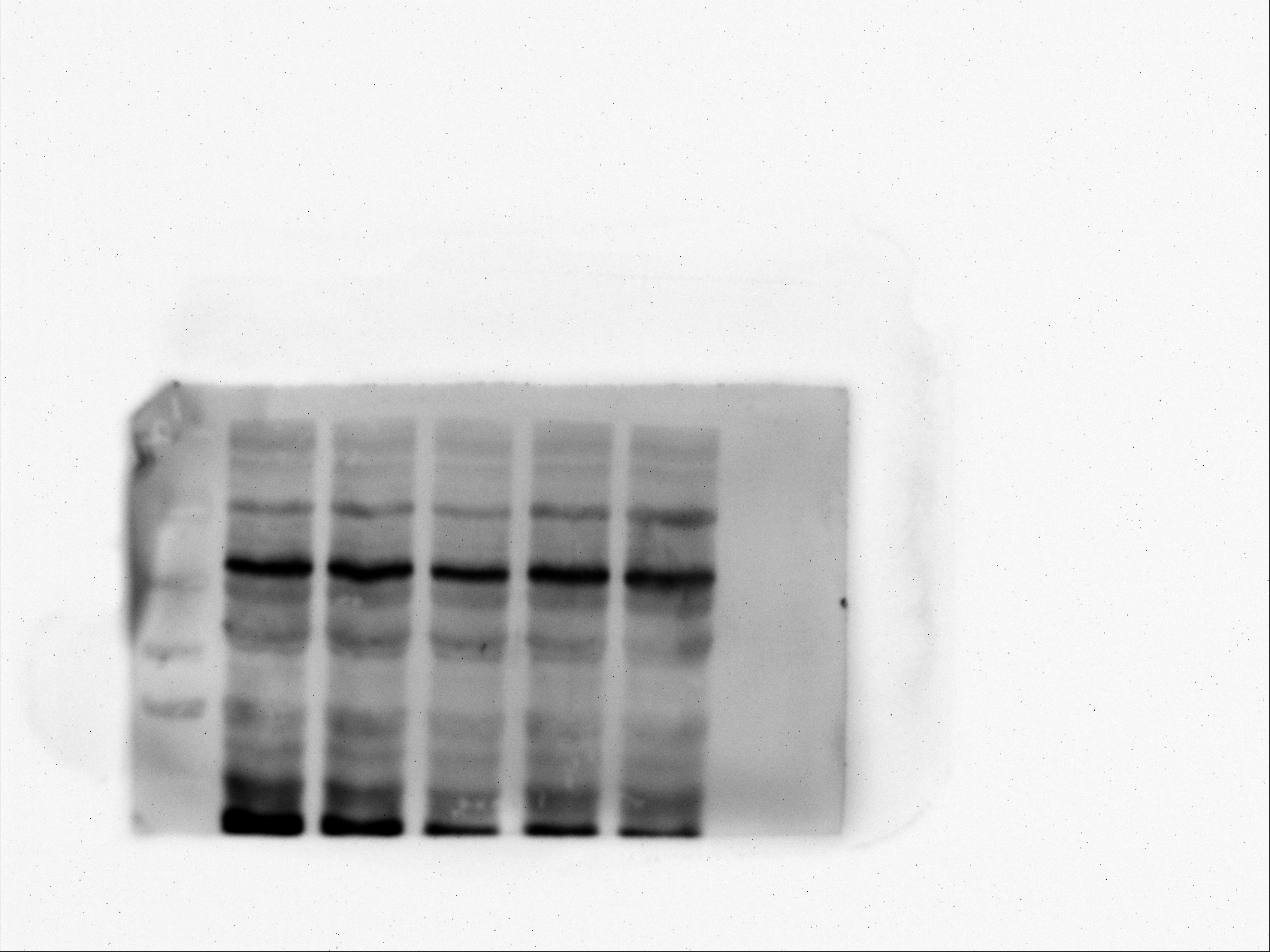

Supplement: Supplementary file 3 — Supplementary Figure 3. [file 41598_2024_57852_MOESM3_ESM.tif]

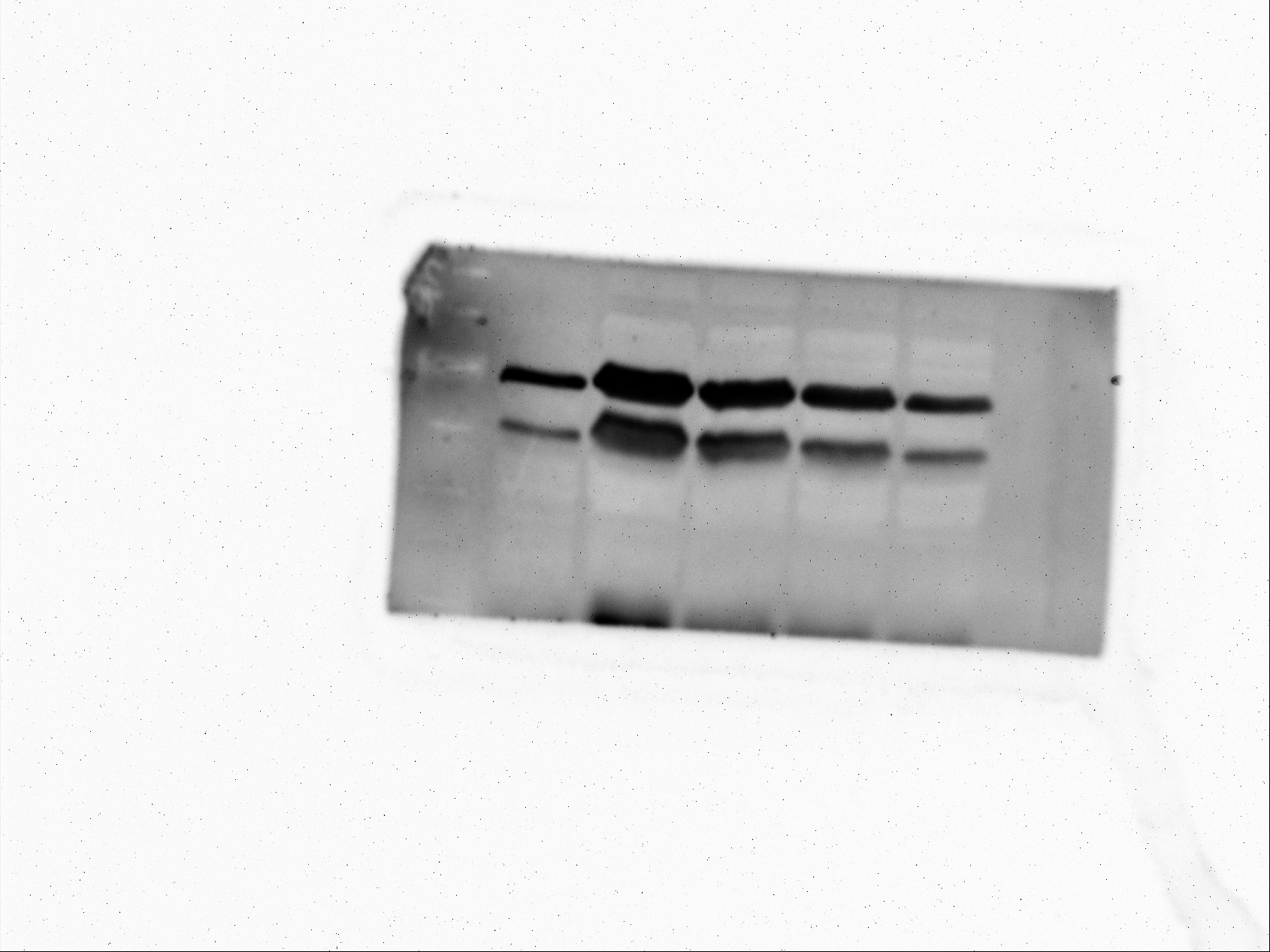

Supplement: Supplementary file 4 — Supplementary Figure 4. [file 41598_2024_57852_MOESM4_ESM.tif]
